# Molecular dynamics of photosynthetic electron flow in a biophotovoltaic system

**DOI:** 10.1016/j.ese.2024.100519

**Published:** 2024-12-15

**Authors:** Jianqi Yuan, Jens Appel, Kirstin Gutekunst, Bin Lai, Jens Olaf Krömer

**Affiliations:** a Systems Biotechnology Group, Department of Microbial Biotechnology, Helmholtz Centre for Environmental Research - UFZ, 04318, Leipzig, Germany; b Molecular Plant Physiology, University Kassel, 34132, Kassel, Germany; c BMBF Junior Research Group Biophotovoltaics, Department of Microbial Biotechnology, Helmholtz Centre for Environmental Research - UFZ, 04318, Leipzig, Germany

**Keywords:** Mediator, Extracellular electron transport, Flavodiiron protein, Mehler-like reaction, Membrane inlet mass spectrometry

## Abstract

Biophotovoltaics (BPV) represents an innovative biohybrid technology that couples electrochemistry with oxygenic photosynthetic microbes to harness solar energy and convert it into electricity. Central to BPV systems is the ability of microbes to perform extracellular electron transfer (EET), utilizing an anode as an external electron sink. This process simultaneously serves as an electron sink and enhances the efficiency of water photolysis compared to conventional electrochemical water splitting. However, optimizing BPV systems has been hindered by a limited understanding of EET pathways and their impacts on cellular physiology. Here we show photosynthetic electron flows in *Synechocystis* sp. PCC 6803 cultivated in a ferricyanide-mediated BPV system. By monitoring carbon fixation rates and photosynthetic oxygen exchange, we reveal that EET does not significantly affect cell growth, respiration, carbon fixation, or photosystem II efficiency. However, EET competes for electrons with the flavodiiron protein flv1/3, influencing Mehler-like reactions. Our findings suggest that the ferricyanide mediator facilitates photosynthetic electron extraction from ferredoxins downstream of photosystem I. Additionally, the mediator induces a more reduced plastoquinone pool, an effect independent of EET. At very high ferricyanide concentrations, the electron transport chain exhibits responses resembling the impact of trace cyanide. These insights provide a molecular-level understanding of EET pathways in *Synechocystis* within BPV systems, offering a foundation for the future refinement of BPV technologies.

## Highlights

• We develop innovative methods for detailed analysis of photosynthetic electron flow in biophotovoltaic (BPV) systems.
• We reveal the electron source responsible for ferricyanide-mediated extracellular electron transfer (EET) in Synechocystis.
• We clarify that EET compete with flavodiiron proteins for electrons on the photosynthetic electron transport chain.
• We find knocking out the flavodiiron proteins *flv234* improves the specific ferricyanide reduction rate by over 275%.
• We discover that high ferricyanide concentrations induce changes in the electron transport chain independent of EET.

## Graphical abstract

**Figure:**
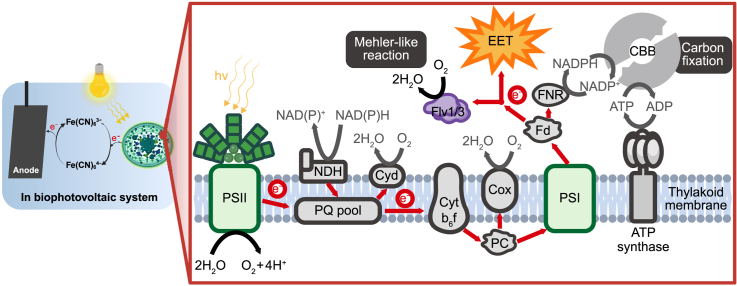

## 1 Introduction

Climate change is jeopardizing the future of humanity, mainly due to the high-carbon-footprint energy used over the past few centuries [1]. Given the growing demand from an increasing global population, developing a sustainable approach to utilizing solar energy is essential [2]. Solar energy is an infinite energy reservoir and is by far the most abundant exploitable resource among all renewable resources [3,4]. Converting energy from sunlight to carbon-free fuel (e.g., hydrogen) is one of the most promising methods for reaching climate targets [5]. The primary challenge lies in producing hydrogen sustainably. Over billions of years, the natural oxygenic photosystem (in cyanobacteria, microalgae, and plants) has evolved into a highly efficient mechanism for capturing solar energy. This system absorbs sunlight with a quantum efficiency of over 30% and splits water into oxygen, protons, and electrons. These protons and electrons are ideal for hydrogen production.

Biophotovoltaics (BPV) is a new technology that has been developed to achieve this goal. BPV uses oxygenic photoautotrophic microbes as efficient catalysts to split water molecules using sunlight in an electrochemical cell. The photosynthetic electrons produced are harvested via electrodes and used for biomass formation [6]. Specifically, photosystem II (PSII) generates electrons through water splitting, which then travel through the photosynthetic electron transport chain, involving plastoquinone (PQ), the cytochrome *b*_6_f complex (Cyt *b*_6_f), plastocyanin (PC), photosystem I (PSI), and ferredoxin, eventually converting to NADPH for carbon fixation. In a BPV system, some electrons can be extracted and collected by extracellular electrodes, known as extracellular electron transfer (EET). The harvested reducing power is then used as electricity or stored as molecular hydrogen. Unlike other (electro-)biotechnological and photochemical technologies, BPV has the advantages of biological systems (i.e., self-assembly, self-maintenance, and self-repair) without depending on organics as the electron source. Moreover, BPV fixes CO_2_ for the biocatalyst regeneration, making it a unique carbon sink approach [7].

In recent years, BPV research has gained increasing attention. A key area of interest is cyanobacteria-powered BPV. For instance, using a printed BPV cell, a previous study produced a sustainable current with the potential to power low-energy devices [8]. The application's potential was further demonstrated when a microprocessor was successfully powered by a BPV system with an aluminum anode under ambient light in a domestic environment for over six months [9]. Process optimization has mainly focused on microbial strains and reactors. For example, cyanobacterial mutants with deactivated terminal oxidases have demonstrated increased electrogenic activity in a BPV system, especially in the dark [7], and optimized BPV reactors and culture media have been developed [[10], [11], [12]]. Among the cyanobacteria employed in BPV research, *Synechocystis* sp. PCC 6803 (hereinafter referred to as *Synechocystis*), a model organism used in photosynthesis research, plays an important role [13,14]. This cyanobacterium is well-characterized, and molecular biology tools are available. However, *Synechocystis* does not produce a particularly high-power output in BPV systems. The power output of these systems is still orders of magnitude lower than those produced by other bioelectrochemical systems based on organic carbon. Moreover, *Synechocystis* does not exhibit significant direct electron transfer in BPV systems. Therefore, redox-active molecules, known as mediators, are necessary to facilitate the transfer of electrons from the cells to the electrode. Various mediator molecules, such as ferricyanide and quinones, have been employed for this purpose [2,6,15]. However, detailed knowledge of the rate-limiting interactions between mediators and cells, which is necessary for the rational optimization of mediated EET in BPV, is still lacking.

Optimizing the mediated EET process requires efficient mediator transport to the electron-carrying complexes in cellular membranes and sufficient metabolic capacity to reduce mediator molecules without leading to dysfunctional redox metabolism in the cells. Optimization is hindered by a lack of knowledge of mediator transport into the periplasm and the mediator's interaction with the electron transfer chain. The disruption or even removal of the cyanobacterial outer cell membrane [16,17] can improve the output of current, highlighting the fact that the low permeability of the outer membrane [17] is currently a limitation of mediator transport. Another limitation may be the cellular capacity to deliver electrons to the mediator. The EET process competes with intracellular metabolic pathways for photosynthetic electrons, possibly leading to low power output [18]. Specifically, the photosynthesis and respiration pathways are closely interlinked on thylakoid membranes via common electron carriers, including the PQ pool, Cyt *b*_6_f, PC, and cytochrome *c*_6_ [19]. Normally, the PQ pool accepts electrons from PSII and the NAD(P)H dehydrogenase complex. However, there are several electron acceptors downstream of the PQ pool. The Calvin–Benson–Bassham (CBB) cycle receives electrons in the form of NADPH downstream of PSI for carbon fixation; cytochrome *c* oxidase (COX) and quinol oxidase (Cyd), known as terminal respiration oxidases, accept electrons from PC/Cyt *c*_6_ and the PQ pool, respectively [19]; and Mehler-like reactions mediated by four flavodiiron proteins (flv1–flv4) consume electrons from ferredoxin downstream of PSI [20]. Mehler-like reactions function as strong photoprotective electron sinks. They can transfer electrons from PSI to O_2_ if there is a risk of photosynthetic electron transport chain over-reduction when the culture conditions change suddenly—for instance, under fluctuating light intensity [21]. In BPV, introducing a mediator as an external electron sink may interfere with these electron transfer networks and induce a new balance among the different electron sinks.

This study systematically investigated the photosynthetic electron flow of *Synechocystis* cultivated in a BPV system using a redox chemical (i.e. the mediator). The mediator has two redox states, i.e. the oxidized form ferricyanide ([Fe(CN)_6_]^3−^) and the reduced form ferrocyanide ([Fe(CN)_6_]^4−^), and is one of the most commonly used mediators in BPV to bridge the electron transfer from the microbial cells to the anode [6,7,22]. Compared to other mediators, this chemical offers advantages such as chemical stability, low biotoxicity, and long-term effectiveness. Several spectrometric methods were used to reveal the redox state of components in the electron transfer chain and to map the corresponding electron flows toward various sinks. The results indicated that EET competed for electrons with Mehler-like reactions downstream of PSI. At high concentrations, the effect of ferricyanide on the electron transport chain was similar to that of trace amounts of cyanide, highlighting the necessity of carefully designing BPV experiments. This work establishes the experimental basis for studying and optimizing mediated electron transfer in BPV systems using cyanobacteria as biocatalysts.

## 2 Materials and methods

### 2.1 Strains and cultivation

*Synechocystis* sp. PCC 6803 was obtained from the Pasteur Culture Collection of Cyanobacteria (Paris, France). To reveal the relationship between EET and Mehler-like reactions, three additional flavodiiron protein–mutant strains were tested in a multi-cultivator (compare Section 2.2.2) for ferricyanide reduction tests: *Synechocystis* Δ*flv*3 (deficient in protein flv3), *Synechocystis* Δ*flv*24 (deficient in flv2 and flv4), and *Synechocystis* Δ*flv*234 (deficient in flv2, flv3, and flv4). These mutant strains have been described elsewhere [23].

All strains were maintained in a buffered BG11 medium [24] with 8% dimethyl sulfoxide (DMSO) at −80 °C. For reactivation, cells were plated on BG11 agar plates and incubated in a photo incubator (SE-41, Percival Scientific, USA) for about one week at 30 °C and 75% relative humidity under 50 μmol photons m^−2^ s^−1^. Subsequently, colonies from the plates were inoculated into baffled shake flasks containing 50 mL of buffered BG11 medium. A 10 mM solution of 4-(2-hydroxyethyl)-1-piperazine ethanesulfonic acid (HEPES) was utilized as a buffer, with the pH adjusted to 7.5 by adding 5 M NaOH. Liquid cultures were incubated in a photo incubator (Multitron Pro; Infors, Bottmingen, Switzerland) at a rotational speed of 150 rpm with a 25 mm orbital throw, maintained at a temperature of 30 °C and a relative humidity of 75%. The cultures were placed under ambient CO_2_ conditions and exposed to continuous white light at an intensity of 50 μmol photons m^−2^ s^−1^. After six days of cultivation, the culture reached an optical density (OD_750_) of about 3.5, and the cells were harvested by centrifugation (6000 g, 5 min, room temperature (RT)). The supernatants were decanted, and the cell pellets were resuspended in a fresh medium and used for inoculation. Wild-type (WT) *Synechocystis* was inoculated into BPV reactors for pulse-amplitude modulation (PAM) and Dual/KLAS-NIR tests. WT *Synechocystis* and the three mutant strains were used in ferricyanide reduction tests in multi-cultivators.

### 2.2 Reactors

#### 2.2.1 BPV reactors

The BPV reactors were set up and operated as described previously [11,25]. The working chamber, with a volume of approximately 270 mL, contained a carbon cloth (1071HCB; Fuel Cell Store, USA) serving as the working electrode, with a projected surface area of 12.5 cm^2^. To enhance hydrophilicity and clean the carbon cloth surface, the cloth was pretreated by incubation in a 2 mM cetrimonium bromide solution at 40 °C for 16 h. A 3 × 7 cm steel mesh (FE6210; Advent Research Materials, UK) was used as the counter electrode, and Ag/AgCl/KCl_sat_ was used as the reference electrode (RE-1CP; Als, Japan). The working and counter electrodes were separated with a circular cation exchange membrane 9 mm in diameter (CMI-7000; Membranes International, USA). The shortest distance between the working and reference electrodes was about 2 cm. The temperature was maintained at 30 °C using a recirculating thermostat and a water jacket. Cell precipitation was prevented by magnetic stirring at 400 rpm. Air flow was provided at 20 mL min^−1^ using rotor flowmeters (B3HT S1, InFlux, UK).

Some system modifications were made specifically for this study. First, a membrane inlet mass spectrometry (MIMS) probe was inserted into the working chamber (Supplementary Material Fig. S1). Second, a porous, autoclavable polytetrafluoroethylene filter with a 10 μm pore size (F765-56; Bola, Germany) was introduced into the system to sparge gases. This filter can enhance effective aeration and save gases during MIMS tests. The light was provided with a light-emitting diode (LED) jacket carrying 45 red LEDs (Conrad Electronic, Germany) at 100 μmol photons m^−2^ s^−1^ (0.16 W per LED), eliminating blue light absorption by ferricyanide as a confounding factor.

Utilizing sterile syringes, the inoculum was introduced into the BPV reactors, filled with BG11 medium exhibiting a conductivity of 4.01 mS cm^−1^. This procedure aimed to achieve a starting OD_750_ of approximately 0.6 within a total culture volume of 250 mL. BPV cultures were then performed for five days. The current was monitored using a potentiostat (VMP3; BioLogic, USA). BPV experiments were conducted to monitor growth, electron transfer, and dissolved gases. Three reactors were used in four independent runs. Reactor 1 contained 0.5 mM ferricyanide (oxidized form) as the mediator, with a potential of +0.5 V (vs. Ag/AgCl) between the working and reference electrodes. This setup allowed the oxidation of ferrocyanide (reduced form) to ferricyanide at the anode, facilitating EET. Reactor 2 contained 0.5 mM ferrocyanide, with 0 V (vs. Ag/AgCl) bias. This maintained the mediator in its reduced form, preventing EET. Reactor 3 contained no mediator, and no potential was applied. The effect of EET was investigated by comparing the performance of the first two reactors. The results were then compared with those of the blank reactor to gain insights into the non-EET effects of adding a mediator.

#### 2.2.2 Multi-cultivators

To test the influence of the flavodiiron proteins on mediator reduction, WT *Synechocystis*, *Synechocystis* Δ*flv*3, *Synechocystis* Δ*flv*24, and *Synechocystis* Δ*flv*234 were inoculated into 80 mL cultures at an OD_750_ of 0.2 in a multi-cultivator (MC 1000-OD; Photon Systems Instruments, Czech Republic). Each strain was cultivated in triplicate for four days under continuous warm white light at 100 μmol photons m^−2^ s^−1^. The temperature was maintained at 30 °C, and aeration was set to 1 vvm. The medium contained 0.5 mM potassium ferricyanide. To measure OD_750_, 1 mL samples were taken every 24 h.

### 2.3 Analytics

#### 2.3.1 Cell density

To assess growth, 2 mL of culture was extracted from the flask or reactor on a daily basis. Cell density was monitored as OD_750_ using a spectrophotometer (Libra S11; Biochrom, UK) and water as a blank. Cell numbers were measured using a Multisizer 3 (Beckman Coulter, USA). Chlorophyll *a* (Chl_a_) concentrations were measured spectrophotometrically at 470, 665, and 720 nm following the 4 °C methanol extraction method [26], and the sample pH was determined using a pH meter (SevenCompact S220; Mettler Toledo, Switzerland).

#### 2.3.2 Mediator concentration

The cells were completely removed by centrifugation at 17,000*g* for 5 min, and absorbance at 420 nm was used to estimate the ferricyanide concentration based on formula 1 abs = 1.02 mM^−1^ cm^−1^ [13].

#### 2.3.3 Mass spectrometry for gas analysis

MIMS was used to analyze CO_2_ uptake, O_2_ formation, and ^16^O and ^18^O labeling, as described below. An HPR-40 system (Hiden Analytical, UK) equipped with an eight-way multi-stream selector (HPR-40 Multi-Probe inlet system) was connected to each BPV reactor using stainless steel MIMS probes (500 mm standard direct membrane inlet probes; Hiden Analytical, UK). This setup allowed online measurements of the dissolved gases directly in the liquid in the working electrode chamber of the BPV system.

#### 2.3.4 Determination of carbon fixation rates

The carbon fixation rate of *Synechocystis* was determined by MIMS following a previously described method [27] with the following modifications. Prior to the inoculation of the BPV reactor, the centrifuged pellet was washed with fresh medium containing 1.5 mM NaHCO_3_. This procedure was implemented to prevent carbon starvation, which has the potential to induce a rapid decline in the level of dissolved inorganic carbon in the medium immediately following inoculation. *Synechocystis* was then cultivated for 30 min in the BPV system in complete darkness, with an aeration of 20 cm³ min⁻^1^, while MIMS was started using the secondary electron multiplier (SEM) detector (amplifier voltage of 1050 V, normal dwelling and settling speed). After 30 min, the CO_2_ signals reached stability. Subsequently, the light-emitting diodes were activated, the aeration process was ceased, and the reactor was securely sealed. CO_2_ concentration changes were dynamically monitored until a stable bottom signal was reached (after about 90 min). At a stable temperature and pH, the dissolved CO_2_ concentration is proportional to the total C_*i*_ concentration in the system and can thus be used for calculations [28]. At a very low external C_*i*_ concentration, C_*i*_ uptake is limited by the carbon concentration mechanism, whereas at high concentrations, C_*i*_ uptake is limited by carbon fixation [27]. In our case, the different kinetics of C_*i*_ limitation by carbon fixation and carbon concentration mechanism were distinct (Supplementary Material Fig. S2). This allowed the calculation of the carbon fixation rate based on the high C_*i*_ concentration data.

#### 2.3.5 Determination of respiration rates

Following the carbon fixation rate measurement method, air was bubbled into the illuminated system for 10 min. The system was then resealed, and the light was switched off. The oxygen uptake rate was obtained by MIMS monitoring for another 20 min. The rate measured immediately after illumination is closest to the real rate under illumination [29]. The rate was calculated according to equation (1), and the final result was normalized to Chl_a_ content and given as μM O_2_ per h per μM Chl_a_.

(1) $$\text{Respiration}\;\text{rate}=\frac{C_{O_{2}}\left(t_{0}\right)-C_{O_{2}}\left(t_{i}\right)}{t_{i}-t_{0}}\times \frac{1000\times 60}{32\times C_{{\text{Chl}}_{a}}}$$

where *t*_0_ and *t*_*i*_ represent the monitoring start and end times, respectively; $C_{O_{2}}$ denotes the O_2_ concentration (in mg L^−1^) at the measurement time point (in minutes), and $C_{{\text{Chl}}_{a}}$ is the Chl_a_ concentration.

#### 2.3.6 Photosynthetic oxygen exchange

During illumination, oxygen evolves from water splitting, and some oxygen is simultaneously consumed in respiration, photorespiration, and Mehler-like reactions. These concurrent processes can be estimated using isotopically labeled oxygen. Measurements and calculations of ^16^O_2_ and ^18^O_2_ were performed as described previously [[30], [31], [32]]. Initially, MIMS was employed to monitor N_2_, ^16^O_2_, and ^18^O_2_ while the system ran in the dark after inoculation. After about 30 min, the system was sparged with N_2_ until the ^16^O_2_ concentration reached approximately half the atmospheric level. Subsequently, ^18^O_2_ (>97% ^18^O_2_ content; Eurisotop, Germany) was sparged into the system at 10 cm^3^ min^−1^ until the ^18^O_2_ concentration surpassed that of ^16^O_2_. N_2_ was then sparged again for a short period to remove ^18^O_2_ accumulated in the headspace and accelerate the gas–liquid phase equilibrium of the dissolved gases. After about 1 h, all gases reached relatively stable concentrations. The system was then sealed, illumination was turned on, initiating oxygen evolution and consumption. The ^16^O_2_ and ^18^O_2_ concentrations were monitored for about 30 min. Based on the variations in these two concentrations, the oxygen evolution and uptake rates were deconvoluted and calculated according to the following equations [33]:

(2) $${O_{2}}_{\text{uptake}}\;\left[\text{mg}\;L^{-1}\;\min^{-1}\right]=\frac{C_{18_{O_{2}}}\left(t_{0}\right)-C_{18_{O_{2}}}\left(t_{i}\right)}{t_{i}-t_{0}}\left(1+\frac{C_{16_{O_{2}}}\left(t_{0}\right)}{C_{18_{O_{2}}}\left(t_{0}\right)}\right)$$

(3) $${O_{2}}_{\text{evolution}}\left[\text{mg}\;L^{-1}\;\min^{-1}\right]=\frac{C_{16_{O_{2}}}\left(t_{i}\right)-C_{16_{O_{2}}}\left(t_{0}\right)}{t_{i}-t_{0}}-\frac{C_{18_{O_{2}}}\left(t_{0}\right)-C_{18_{O_{2}}}\left(t_{i}\right)}{t_{i}-t_{0}}\left(1+\frac{C_{16_{O_{2}}}\left(t_{0}\right)}{C_{18_{O_{2}}}\left(t_{0}\right)}\right)$$

where $C_{16_{O_{2}}}$ and $C_{18_{O_{2}}}$ represent the respective concentrations (in mg L^−1^) of ^16^O_2_ and ^18^O_2_, respectively, at the measurement time point (in minutes), and *t*_0_ and *t*_*i*_ denote the monitoring start and end times, respectively. The reactors had about 50 mL of headspace. In the exchange rate and kinetics calculations, the gas concentration changes in the headspace were compensated for according to Henry's law (30 °C, 1 atm).

#### 2.3.7 State transition measurement

State transitions were analyzed using multicolor PAM (Walz, Germany) according to Appel et al.‘s [34] method, with the following modifications. The built-in red LED was used as both the measuring and actinic light, and the measuring light intensity and gain were set to 1. Because the aim was to estimate the effects of mediators and KCN on state transition, different concentrations of ferricyanide, ferrocyanide, and KCN were added during the measurement instead of glucose. Initially, to determine F_0_, the measuring light was turned on, and a saturated pulse at an intensity of 3500 μmol photons m^−2^ s^−1^ was applied to a cuvette filled with 1.2 mL of *Synechocystis* culture at a cell density of 2.5 μg Chl_a_ mL^−1^. Subsequently, the red actinic light illuminated the sample at an intensity of 110 μmol photons m^−2^ s^−1^ for 3 min, followed by another saturated pulse to obtain Fm^−^. During the following 30 s, 63.2 μL of a twenty-fold concentrated solution of the substance to be tested (oxidized/reduced mediator, different concentrations of KCN, and H_2_O as a blank control) was added to the cuvette in the dark. After another round of illumination with red actinic light for 3 min, a saturated pulse was applied to obtain Fm^+^. The state change was estimated using the following equation: (Fm^−^ − Fm^+^)/F_0_.

#### 2.3.8 Chlorophyll fluorescence emission spectroscopy at 77 K

Triplicate measurements were performed after the BPV system was operated for 3 h. Samples were subsequently transferred into sampling bottles that were provided with identical illumination. Glass tubes featuring an outer diameter of 7 mm, an inner diameter of 5 mm, and a length of 18 cm were utilized as pipettes for the aspiration of the liquid. The tubes were snap-frozen in liquid nitrogen and kept at −80 °C until the measurement. During the measurement, the tubes were held in a dewar containing liquid nitrogen (77 K, equals to −196 °C) inside a fluorescence spectrophotometer (F-2700; Hitachi, Japan) as described previously [23]. A wavelength of 580 nm was used to excite the phycobilisomes, and a wavelength of 440 nm was used to excite Chl_a_. As a background sample, a cell-free medium from the same time point was obtained by filtering out the cells and snap-frozen. The measured background signals were then subtracted from the target signals. The efficiency of energy transfer from phycobilisomes to PSI was calculated as $\frac{F_{\text{PSI}}\left(580\right)}{F_{\text{PSI}}\left(440\right)}$ [35].

#### 2.3.9 Dual/KLAS-NIR measurement

The absorption of P700, PC, and ferredoxin was quantified using Dual/KLAS-NIR (Walz, Germany). Differential model plots used for the deconvolution of the P700, PC, and ferredoxin signals [36] were obtained following a previously described method [23]. A 1.2 mL solution containing *Synechocystis* at a final cell density of 20 μg Chl_a_ mL^−1^ was measured in a cuvette at room temperature with the addition of an oxidized/reduced mediator or different concentrations of KCN. Four wavelength pairs were balanced for each sample, and calibration was performed. Data were then recorded for 3 s. After 1 s, a red-light pulse at an intensity of 1350 μmol photons m^−2^ s^−1^ was applied for 600 ms. The final plot represented the averages of 16 measurements, which were separated by 30 s periods of darkness.

## 3 Results and discussion

### 3.1 Effects of EET on cell growth, carbon fixation, and respiration

**Fig. 1:**
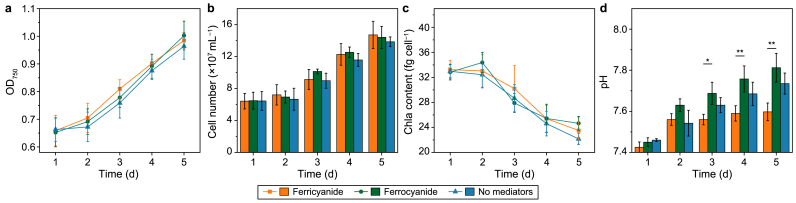
Growth profiles of *Synechocystis* cultivated under different conditions in the BPV system for five consecutive days. **a**, OD_750_; **b**, cell number; **c**, Chl_a_ content per cell; **d**, pH value variation. Samples were taken and measured daily around the same time. In all figures, ‘ferricyanide’ represents the BPV reactors with 0.5 mM ferricyanide addition as well as +0.5 V (vs. Ag/AgCl) potential bias applied, ‘ferrocyanide’ represents the BPV reactors with 0.5 mM ferrocyanide addition and 0 V (vs. Ag/AgCl) bias applied. As the blank, ‘no mediators’ indicates the reactors without a mediator added and no potential bias. Means and standard deviations are presented (*n* = 4). The asterisk indicates a statistically significant difference to the ‘ferrocyanide’ group based on ANOVA (∗*P* ≤ 0.05, ∗∗*P* ≤ 0.01).

An initial characterization was performed to assess the influence of EET on cell growth by comparing the cases of reduced and oxidized mediators, which represented the largest metabolic electron sinks in the photosystems. EET was possible only under oxidized mediator conditions, with photocurrent generated after inoculation and lasting five days (Supplementary Material Fig. S3). There were no significant differences in OD_750_, cell numbers, or Chl_a_ contents between the oxidizing and reducing mediator conditions or in the absence of either a mediator or electrode bias (Fig. 1a–c). Contrary to an earlier study reporting impaired growth in the presence of ferricyanide or quinones in BPV systems [37], no growth differences were observed with or without a mediator. The only significant difference was in the medium's pH, with oxidizing conditions leading to a more stable pH (Fig. 1d).

From a reactor engineering perspective, a BPV system also offers a feasible solution to the issue of alkalization in phototrophic processes. Normally, a limitation of charge transfer across the ion exchange membrane often leads to a decreasing pH in the anode chamber [38,39]. However, cyanobacteria are known to alkalize the culture medium by consuming CO_2_ [40,41] and to produce considerably smaller quantities of organic acids than heterotrophs. Overall, in our BPV system, charge transfer across the ion exchange membrane seemed well balanced, without external pH control (Fig. 1d). This holds promise for creating a relatively inexpensive reactor design and achieving stable operation.

**Fig. 2:**
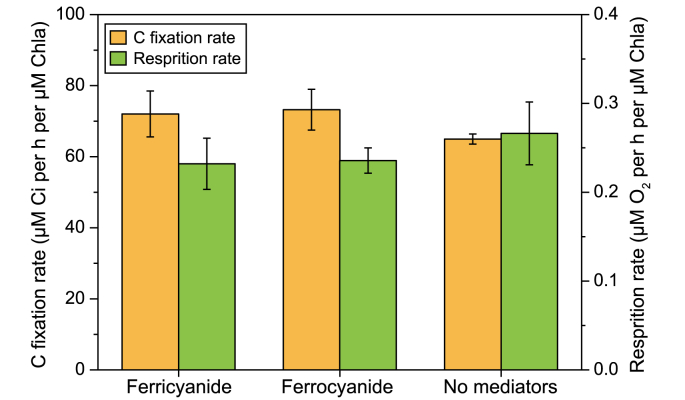
The activity of two-electron sinks of *Synechocystis* cultivated under different conditions in BPV systems. In both plots, ‘ferricyanide’ represents the BPV reactors with 0.5 mM ferricyanide addition as well as +0.5 V (vs. Ag/AgCl) potential bias applied, ‘ferrocyanide’ represents the BPV reactors with 0.5 mM ferrocyanide addition and 0 V (vs. Ag/AgCl) bias applied. As the blank, ‘no mediators’ indicates the reactors without a mediator added and no potential bias. Means and standard deviations are presented (*n* = 4). No significant differences based on ANOVA (*P* ≤ 0.05) could be observed.

The absence of a clear growth difference between the EET and control conditions suggests that the magnitude of EET was insufficient to effectively compete for electrons with carbon fixation [6]. Indeed, an analysis of carbon uptake showed that the differences in carbon fixation were nonsignificant (ANOVA; *P* = 0.8182; Fig. 2). As the most dominant sink for linear electron transport downstream of PSI, the CBB cycle typically consumes the vast majority of electrons [42]. This suggests that, under certain conditions, there may be considerable potential for redirecting electrons to the electrode. This also explains the quite similar carbon fixation rates of the three reactors. The respiration rates of the cells in the three systems were also comparable. Even though respiration activity is low under illumination, with only about 6% of electrons flowing to O_2_ attributed to respiration [43], it is still a crucial electron sink on thylakoid membranes, as it helps to maintain redox balance and accommodate sudden light changes [7,19]. The fact that the mediator did not interfere with respiratory processes may also be promising for ensuring BPV robustness during dark periods.

### 3.2 Relationship between EET and mehler-like reactions

**Fig. 3:**
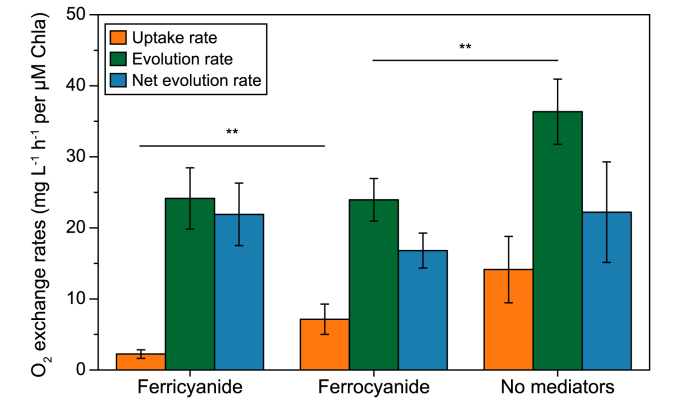
Photosynthetic oxygen exchange of *Synechocystis* cultivated under different conditions in BPV systems. Under illumination, O_2_ evolves from water splitting by photosystem II (PSII), while O_2_ is simultaneously consumed by Mehler-like reactions. Net evolution rate is the difference between evolution and uptake rate. In both plots, ‘ferricyanide’ represents the BPV reactors with 0.5 mM ferricyanide addition as well as +0.5 V (vs. Ag/AgCl) potential bias applied, ‘ferrocyanide’ represents the BPV reactors with 0.5 mM ferrocyanide addition and 0 V (vs. Ag/AgCl) bias applied. As the blank, ‘no mediators’ indicates the reactors without a mediator added and no potential bias. Averages and standard deviations (*n* = 4) are given for 30 min. Asterisks indicate statistically significant differences to the ‘ferrocyanide’ group based on ANOVA (∗*P* ≤ 0.05, ∗∗*P* ≤ 0.01).

The net O_2_ evolution rate (Fig. 3) suggested that the introduction of mediators had little influence on the photosynthetic electron transfer chain. However, a more detailed analysis of O_2_ evolution and consumption showed that the mediators significantly affected the photosynthetic electron transfer chain. MIMS and ^18^O_2_ were used in combination to distinguish the O_2_ evolution rate from the O_2_ uptake rate (Fig. 3). The photosynthetic efficiency, represented by the O_2_ evolution rate, dropped sharply from 36.3 to approximately 24 mg L^−1^ h^−1^ per μM Chl_a_ with the addition of either ferricyanide or ferrocyanide. This decline was independent of the mediators’ redox state, suggesting that the presence of the mediators, rather than the charge transfer to the electrode, caused the drop. A similar effect has also been observed in chloroplast studies in which photosynthesis and O_2_ evolution were inhibited by the addition of ferricyanide [44,45]. It is widely assumed that the metabolism of *Synechocystis* is highly flexible and rarely operates at maximum photosynthetic capacity [46]. Increasing the electron demand, such as by adding nitrate as an extra sink, can even boost photosynthetic activity [46]. However, this does not apply to the introduction of EET as an extracellular electron sink. In this study, EET did not affect photosynthetic activity at all, but the O_2_ uptake rate differed significantly (ANOVA; *P* = 0.0088) between the BPV reactors with ferricyanide (EET possible) and those with ferrocyanide (no ETT possible). The presence of an oxidized mediator and active EET led to a considerably lower oxygen uptake rate (Fig. 3). In *Synechocystis*, two main metabolic processes consume O_2_ along the electron transport chain: respiration mediated by terminal oxidases and Mehler-like reactions catalyzed by flavodiiron proteins. Respiration is usually severely limited under illumination [47]. In a previous study, electron flow to O_2_ was as high as 40% of that leaving PSII, whereas only 6% could be attributed to respiration under illumination at an intensity of 70 μmol photons m^−2^ s^−1^ [43]. It has also been shown that respiration does not play a role in *Synechocystis* Δ*flv*1–Δ*flv*3 under illumination in various intensities [47]. Therefore, O_2_ consumption under illumination can be attributed to Mehler-like reactions. The significant changes in the O_2_ uptake rate in our BPV systems could thus be attributed to variations in Mehler-like reactions. Since the only difference between the ferricyanide and ferrocyanide experiments was active EET, it can be concluded that ferricyanide-mediated electron transfer inhibits Mehler-like reactions. This is an important finding since these reactions are important valves for an electron surplus under high-intensity illumination, basically allowing cells to balance excessive water splitting. *Synechocystis* has four isoforms of flavodiiron proteins, flv1–flv4, with flv1/flv3 and flv2/flv4 acting as two kinds of hetero-oligomers. The former are responsible for a transient but strong O_2_ photoreduction under fluctuating illumination, whereas the latter contribute to Mehler-like reactions more slowly and steadily under low illumination [20,21,48]. Compared to flv2 and flv4, flv1 and flv3 are considered more fundamental O_2_ consumers [21,32]. It has been shown that flv1 and flv3 accept electrons downstream of PSI and use ferredoxin as the donor [20,21,48], whereas this remains an open question in the case of flv2 and flv4.

**Fig. 4:**
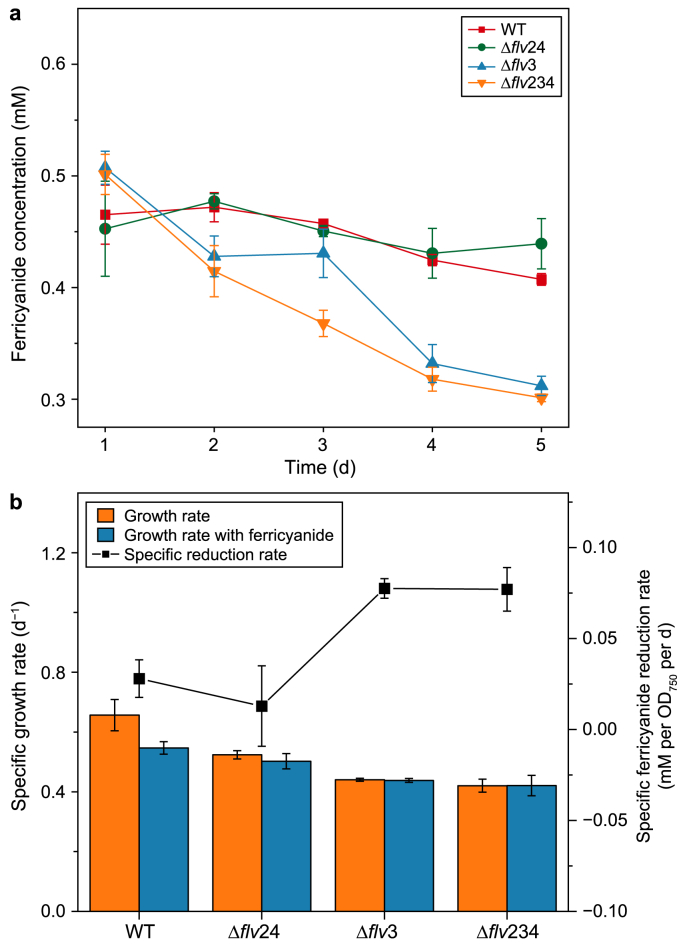
Growth and ferricyanide reduction performance of four *Synechocystis* strains cultivated in multi-cultivator vessels: wild-type *Synechocystis* (WT), *Synechocystis* lacking protein flv2 and flv4 (Δ*flv*24), *Synechocystis* lacking protein flv3 (Δ*flv*3), and *Synechocystis* lacking protein flv2, flv3 and flv4 (Δ*flv*234). **a**, Ferricyanide reduction performance of four strains cultured with initial 0.5 mM ferricyanide addition during four days' cultivation. **b**, Specific growth and ferricyanide reduction rates. In both plots, the error bars stand for the standard deviation over three replicates (*n* = 3).

**Fig. 5:**
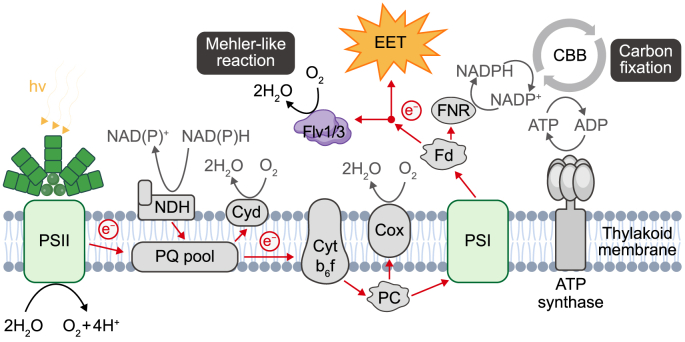
Schematic diagram of the electron transfer chain in the thylakoid membrane of *Synechocystis* and the electron transport routes affected by ferricyanide-mediated extracellular electron transfer (EET). Red arrows represent the electron transport pathway. In a ferricyanide-mediated BPV system, once EET starts acting, no obvious influence on the chain can be detected apart from decreasing the rate of Mehler-like reactions. EET competes for electrons with the flavodiiron protein flv1/3 (Flv 1/3). The redox potentials of key components, measured against the standard hydrogen electrode (SHE), are as follows: P680 in photosystem II (PSII), 1.19 V; excited P680, −0.62 V; plastoquinone (PQ), 0.08 V; cytochrome *b*_6_f complex (Cyt *b*_6_f), 0.3 V; plastocyanin (PC), 0.37 V; P700 in photosystem I (PSI), 0.42 V; excited P700, −1.3 V; ferredoxin (Fd), −0.41 V; ferredoxin-NADP^+^ reductase (FNR), −0.38 V. The redox potential of ferricyanide in neutral aqueous conditions is 0.42 V. Abbreviations: Calvin-Benson-Bassham cycle (CBB), NADH dehydrogenase (NDH), cytochrome *c* oxidase (Cox), cytochrome bd quinol oxidase (Cyd).

To confirm this role of EET in *Synechocystis*, ferricyanide reduction tests with different mutant strains were performed. *Synechocystis* Δ*flv*3, *Synechocystis* Δ*flv*24, and *Synechocystis* Δ*flv*234 grew slower than WT *Synechocystis*. The addition of ferricyanide did not significantly affect the growth of the mutant strains (Fig. 4b and S4). However, the ferricyanide reduction rates of *Synechocystis* Δ*flv*3 and *Synechocystis* Δ*flv*234 were considerably higher than those of WT *Synechocystis* and *Synechocystis* Δ*flv*24 (Fig. 4a and b). These results indicate that the presence of the mediator and EET rerouted electrons from flv1 and flv3, since *Synechocystis* Δ*flv*3 and *Synechocystis* Δ*flv*234 lack this flavodiiron complex. However, EET did not have the capacity to replace flv2 and flv4. The mediator most likely shared the same electron source as flv1 and flv3, suggesting that it may have the capacity to replace this natural photoprotective system (Fig. 5). This means that cells could use the BPV as an electron valve under high illumination, although the electron transfer rate is currently low. Moreover, EET could be enhanced by eliminating flv1 and flv3. Furthermore, BPV efficiency is likely to improve under stronger illumination, as it will generate excess electrons that are typically processed by flavodiiron proteins, thereby increasing the electrons available for EET. We recommend testing this approach in future studies.

In short, the EET pathway using ferricyanide can compete for electrons with the flavodiiron proteins flv1 and flv3 involved in Mehler-like reactions while not affecting carbon fixation, net O_2_ evolution, or respiration. This indicates that the core function of photosynthesis—converting light energy to chemical energy and subsequent carbon fixation—is unaffected by EET. This finding suggests the possibility of integrating BPV with existing biotechnologies that rely on photosynthetic efficiency, such as biofuel production and carbon capture. Moreover, *Synechocystis* has the potential to be used for electricity generation in BPV systems. This potential could be further explored in experiments under carbon-limiting conditions. With fewer electrons used by the CBB cycle, more could be redirected to the electrode.

### 3.3 Effects of ferricyanide and ferrocyanide on the PQ pool

The finding that EET influenced Mehler-like reactions raised the question of whether this affected electron transfer along the electron transfer chain. To shed light on the oxidation state in the cellular membrane system, the state transitions of *Synechocystis* after the addition of ferricyanide or ferrocyanide were investigated using multicolor PAM fluorometry. State transition in cyanobacteria is a mechanism that allocates the incoming light energy from phycobilisomes to PSII or PSI. This is achieved by the physical movement of phycobilisomes from PSII to PSI or by a movement of the PSII–phycobilisome complex close to PSI, leading to a change in energy transfer (Supplementary Material Fig. S5). It is widely accepted that state transition is regulated by the redox status of the PQ pool. When the PQ pool is oxidized, phycobilisomes direct more energy to PSII (State 1). Conversely, a reduced PQ pool causes phycobilisomes to transfer more energy to PSI (State 2) [49,50]. This process is a rapid adaption mechanism of cyanobacteria, with the shift of phycobilisomes completed within 100 ms [51]. Given that the yield of Chl_a_ fluorescence from PSI is considerably lower than that from PSII in cyanobacteria [52], the fluorescence level should decrease if phycobilisomes transfer more energy to PSI (State 2) when the PQ pool is reduced. In contrast, when the PQ pool is oxidized, phycobilisomes should transfer more energy to PSII, leading to higher fluorescence levels. After normalization to F_0_, the difference between the fluorescence gained by phycobilisome excitation before (Fm^−^) and after (Fm^+^) the addition of a mediator represents the state transition caused by mediator addition. In brief, the higher the ratio of (Fm^−^ − Fm^+^)/F_0_, the greater the decrease in fluorescence when a mediator is added, indicating an active state transition during which phycobilisomes direct more energy to PSI due to a greater reduction in the PQ pool. This approach modifies widely used methods for determining state transition ability [34,53].

**Fig. 6:**
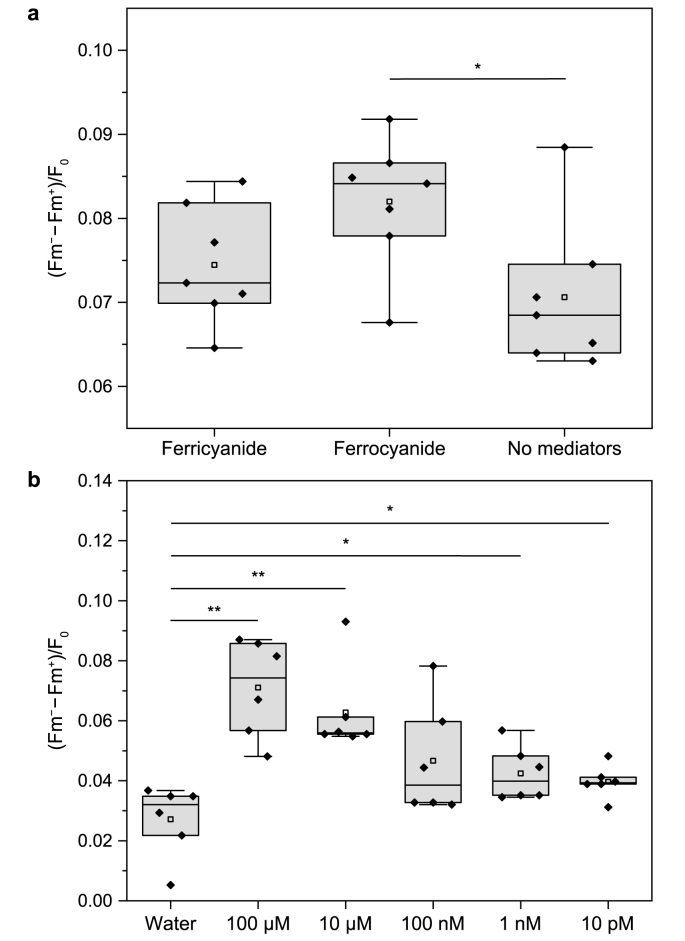
State transition determined by Multi-color pulse-amplitude modulation (PAM). **a**, Box plot of state change after 0.5 mM ferri/ferrocyanide or the same volume of water was added (*n* = 7). **b**, Box plot of state change after different concentrations of KCN or the same volume of water was added (*n* = 6). The black horizontal lines in the boxes represent the median, and the small hollow squares represent the mean. Two separate cultures were used for panels **a** and **b**; hence, the water controls differ, and individual results of each culture should be compared to the respective water control. The asterisk indicates a statistically significant difference to the ‘ferrocyanide’ group based on ANOVA (∗*P* ≤ 0.05, ∗∗*P* ≤ 0.01).

A comparison of the mediators' effects showed a significantly more reduced PQ pool with 0.5 mM ferrocyanide (ANOVA; *P* = 0.0244; Fig. 6a). Furthermore, ferricyanide had a more modest reduction effect than ferrocyanide, probably because EET extracted electrons downstream of the PQ pool. The same conclusion can be drawn from the 77 K fluorescence emission spectra (Supplementary Material Fig. S6). The phycobilisome–PSI efficiency calculated based on the fluorescence spectra also showed that ferricyanide and ferrocyanide led to a more reduced PQ pool and that EET with ferricyanide alleviated the effect to some extent (Supplementary Material Table S1). The greater reduction in the PQ pool was followed by phycobilisomes’ shift from PSII to PSI, with PSII efficiency dropping significantly. This could be the reason for the decrease in the O_2_ evolution rate when ferricyanide or ferrocyanide was present in the system (Fig. 3). Moreover, it can be expected that Mehler-like reactions as a photoprotective mechanism will be more active if no mediator is added and PSII activity increases to maintain redox equilibrium. This may also explain the difference in O_2_ uptake rates between the ferricyanide-containing systems and the mediator-free controls.

The state transition analysis also showed that, like ferricyanide and ferrocyanide, the addition of trace amounts of KCN also reduced the PQ pool. The effect intensified with increasing KCN concentrations (Fig. 6b). KCN is considered a strong inhibitor of the respiratory electron transport chain, as it inactivates respiratory terminal oxidases. A quantity of 5 mM KCN can completely inhibit both O_2_ evolution and O_2_ consumption [17]. KCN can also inactivate PC [54] and possibly even the CBB cycle [55]. In this study, even 100 pM KCN caused a significant state change. This suggests that the downstream sinks of the PQ pool were inhibited to some extent after KCN was added, and this effect led to a more reduced PQ pool.

These results raised the question of whether trace amounts of cyanide were released from the mediator under the given conditions or whether the mediator contained trace amounts of KCN. Under conditions such as those employed in this study, ferricyanide is considered a very stable complex [56]. Indeed, a photometric assay indicated no decrease in total ferricyanide (or ferrocyanide) concentrations. Nevertheless, an additional experiment with higher mediator concentrations was conducted.

### 3.4 Effects of KCN, ferricyanide, and ferrocyanide on PSI

**Fig. 7:**
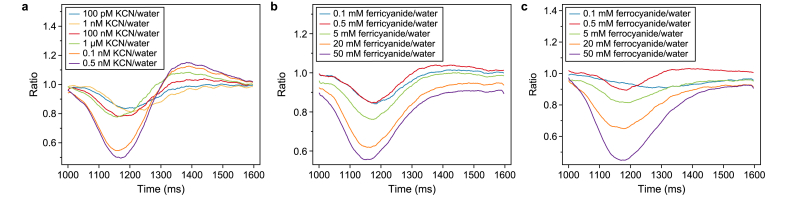
P700 redox status dynamic change of *Synechocystis* lived with different concentrations of KCN and ferro/ferricyanide during 600 ms red light shot. **a**, Normalization of the P700 redox change to the water signal with different concentrations of KCN in addition to that with nothing added during the 600 ms light pulse phase. At 1000 ms, the red actinic light pulse of 1350 μmol photons m^−2^ s^−1^ was applied and lasted for 600 ms. **b**–**c**, Normalization of the P700 changes to the water signal with different concentrations of ferricyanide (**b**) and ferrocyanide (**c**). All measurements were conducted 16 times repeatedly with 30 s darkness intervals; the displayed traces represent the averages.

The dynamic changes in the redox state in the PSI reaction center (P700) at different KCN and mediator concentrations were observed using Dual/KLAS-NIR spectroscopy. During a 600 ms light pulse, the P700 signal showed a strong dip with increasing KCN concentrations (Supplementary Material Fig. S7a). This trend was similar to the P700 signal of a *Synechocystis* mutant strain with COX and Cyd deficiency [23]. After the instantaneous oxidization of P700 when the light was switched on at 1000 ms, P700 received electrons from PSII and was gradually reduced (Phase 1). After about 200 ms, sinks such as flavodiiron proteins located downstream of PSI became relevant, and re-oxidization was observed (Phase 2). During Phase 1, the reduction accelerated with increasing KCN concentrations (Fig. 7a), indicating that KCN inhibited the terminal oxidases in thylakoid membranes, reducing the depletion of electrons.

A comparable effect on P700 was observed in the ferricyanide and ferrocyanide experiments (Supplementary Material Figs. S7b and c). During the P700 reduction phase from 1000 to around 1150 ms, the P700 signal ratio dropped with increasing mediator concentrations (Fig. 7b and c). In other words, the reduction accelerated with increasing mediator concentrations. Moreover, in all three cases, higher concentrations resulted in earlier initiation of re-oxidization. When more than 100 nM cyanide was added, over-oxidization was observed during Phase 2. Both phenomena indicated a donor-side limitation resulting from a drop in PSII efficiency triggered by state transition after a fast P700 reduction. Higher ferricyanide, ferrocyanide, and cyanide concentrations led to a faster reduction in the PQ pool and, consequently, a faster state transition.

The Dual/KLAS-NIR analysis also provided data on the ferredoxin reduction state. The ferredoxin pool was reduced by electrons from PSII when the light pulse was used at 1000–1250 ms (Supplementary Material Fig. S8). The reduction rate increased with increasing cyanide concentrations, indicating that cyanide inhibited terminal oxidases. This led to a more reduced PQ pool, which boosted the electron flux from PSII to PSI. Conversely, unlike KCN, neither ferricyanide nor ferrocyanide showed a clear concentration-dependent trend.

The Dual/KLAS-NIR analysis also showed that the effects of both the reduced and oxidized mediators on the P700 and ferredoxin profiles were similar to that of KCN. The considerable difference in concentrations necessary to cause the observed effects might indicate leakage of trace amounts of CN^−^ from the mediators or the presence of CN^−^ as an impurity in the chemicals used. On the other hand, it could also indicate that both CN^−^ and the mediators affected the same components of the electron transfer chains but with different strengths. This question requires further study. According to previous studies, both ferrocyanide and ferricyanide tend to release toxic free cyanide, especially in the presence of light [[57], [58], [59], [60]]. While ferricyanide may be biotoxic and inhibit oxygen evolution under certain conditions [37,44], it is also used as an anti-caking agent in the food industry [61]. These phenomena highlight that biotoxicity of the mediator is highly condition-dependent. It is important to note that only 0.5 mM mediator was used in the BPV reactors in this work and that photometric mediator quantification indicated no significant loss of mediator. To mitigate these toxic effects, it is essential to determine and use the lowest possible effective concentration of ferricyanide that facilitates EET without causing significant biotoxicity. Exploring alternative, KCN-free mediators could further reduce adverse effects.

## 4 Conclusion

This study employed a sophisticated combination of different measurement tools to shed light on the EET process in BPV systems. The findings suggest that ferricyanide, the most commonly used mediator, can play a dual role in BPV systems. On the one hand, as a mediator, it can serve as an electron acceptor and compete for electrons with the flavodiiron proteins flv1 and flv3 downstream of PSI. On the other hand, it may act on a molecular level independent of the redox state by interacting with individual enzyme complexes of the electron transfer chain, exerting an effect similar to that of trace amounts of KCN. This work provides a quantitative basis for evaluating and optimizing electron harvesting in BPV systems, serving as a formidable instrument for comprehending cellular electron transfer mechanisms.

## CRediT authorship contribution statement

**Jianqi Yuan:** Writing - Review & Editing, Writing - Original Draft, Visualization, Methodology, Formal Analysis, Data Curation. **Jens Appel:** Writing - Review & Editing, Methodology. **Kirstin Gutekunst:** Writing - Review & Editing, Methodology. **Bin Lai:** Writing - Review & Editing, Writing - Original Draft, Supervision, Project Administration, Funding Acquisition, Conceptualization. **Jens Olaf Krömer:** Writing - Review & Editing, Writing - Original Draft, Supervision, Project Administration, Funding Acquisition, Conceptualization.

## Declaration of competing interest

The authors declare that they have no known competing financial interests or personal relationships that could have appeared to influence the work reported in this paper.

The author is an Early Editorial Board Member for *Environmental Science and Ecotechnology* and was not involved in the editorial review or the decision to publish this article.
