# Supplementary material for: Molecular dynamics of photosynthetic electron flow in a biophotovoltaic system
Source: Environ Sci Ecotechnol. 2024 Dec 15;23:100519. doi: 10.1016/j.ese.2024.100519 (PMC11732479; doi:10.1016/j.ese.2024.100519)
Supplement: Multimedia component 1 [file mmc1.docx]

**Supplementary Materials**

**Molecular dynamics of photosynthetic electron flow in a biophotovoltaic System**

**Jianqi Yuan^a^, Jens Appel^b^, Kirstin Gutekunst^b^, Bin Lai*^c^, Jens Olaf Krömer^a^**

^a^ Systems Biotechnology group, Department of Microbial Biotechnology, Helmholtz Centre for Environmental Research - UFZ, 04318 Leipzig, Germany

^b^ Molecular Plant Physiology, University Kassel, 34132 Kassel, Germany

^c^ BMBF junior research group Biophotovoltaics, Department of Microbial Biotechnology, Helmholtz Centre for Environmental Research - UFZ, 04318 Leipzig, Germany


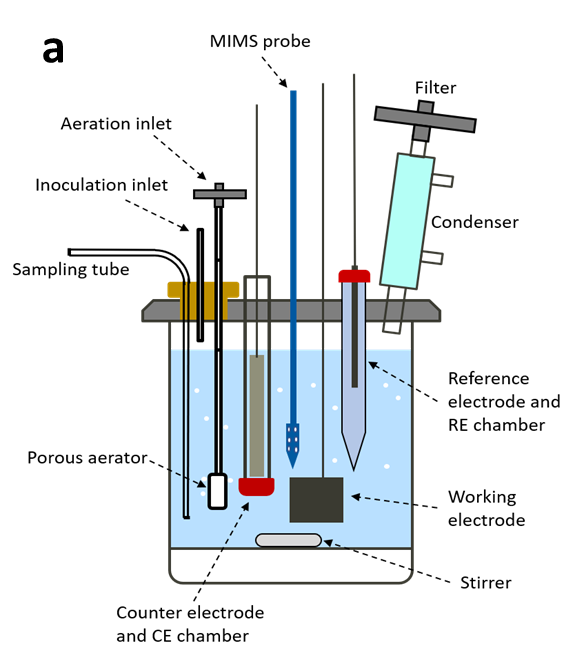

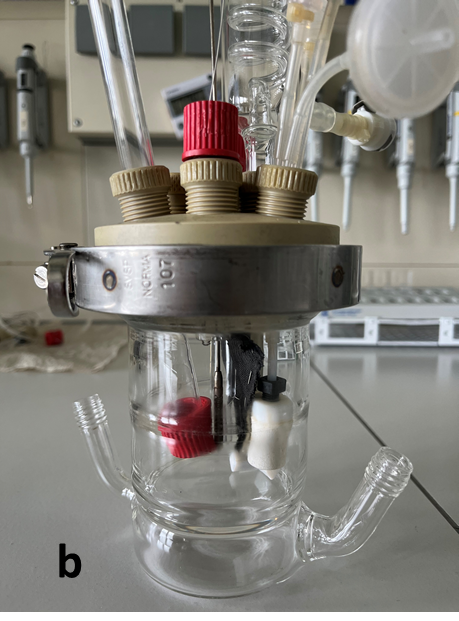

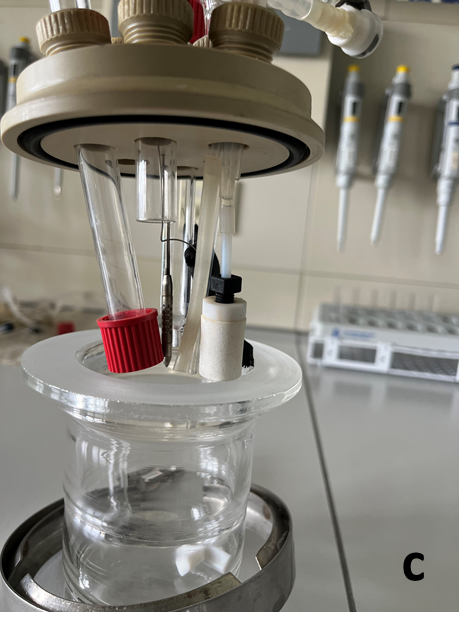


**Figure. S1**. Illustration of BPV reactor. (a) Schematic drawing of BPV system. The reference electrode (RE) chamber was filled with saturated KCl solution inside and communicated with the bulk liquid through a porous glass frit inlaid at the bottom of the chamber. The counter electrode (CE) chamber which is open to the air is filled with sterile BG11 medium, and separated from the working chamber by a proton exchange membrane at the bottom. The sampling tube always connects to a sterile sampling bottle, and the inoculation inlet is sealed up by a cap unless inoculation. Additionally, the whole reactor always stands on a magnetic stirrer controlling the stirrer bar inside the reactor, and is shielded by Faraday box consistently. (b) Photograph of BPV reactor. (c) Photograph of open and disintegrated reactor.


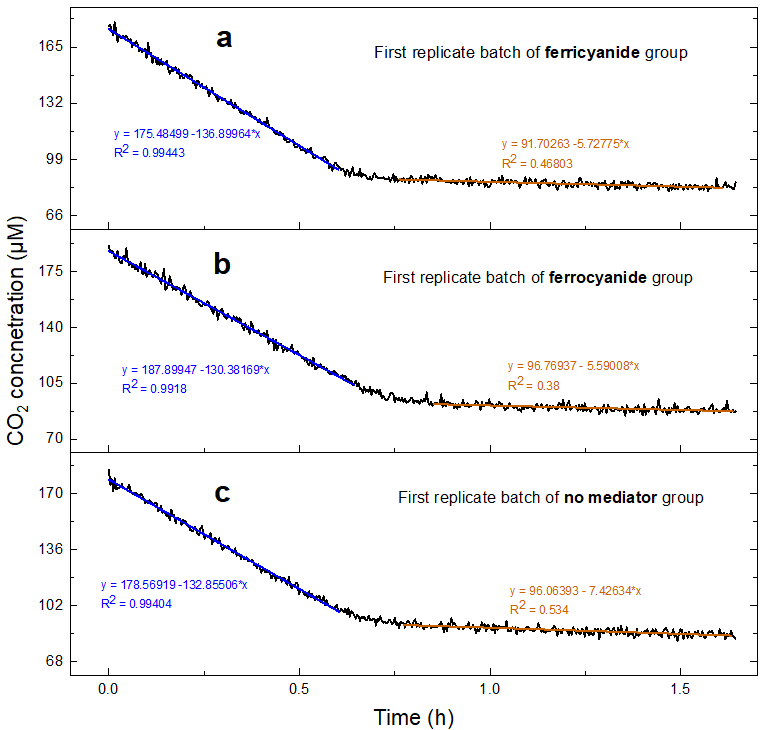


**Fig. S2.** Instances of CO_2_ concentration variation in first replicates of three BPV systems during MIMS measurement for carbon fixation rate determination. (a) CO_2_ dynamic change in the first replicate of BPV system with 0.5 mM ferricyanide addition as well as + 0.5 V (vs. Ag/AgCl) potential bias applied. (b) CO_2_ dynamic change in the first replicate of BPV system with 0.5 mM ferrocyanide addition as well as 0 V (vs. Ag/AgCl) potential bias applied. (c) The change in the first replicate of blank BPV system without ferri/ferrocyanide added or potential bias applied. In all three examples, the boundary between the CBB limited and CCM limited range is quite evident at around 0.7 h. Before that time point, the CO_2_ absorption rate was decided by carbon fixation rate of CBB cycle since the sufficient carbon supply, while it would be decided by CCM when the CO_2_ is deficient after that point. The carbon fixation rates were calculated based on the CO_2_ dynamic change rates in CBB limited phase.


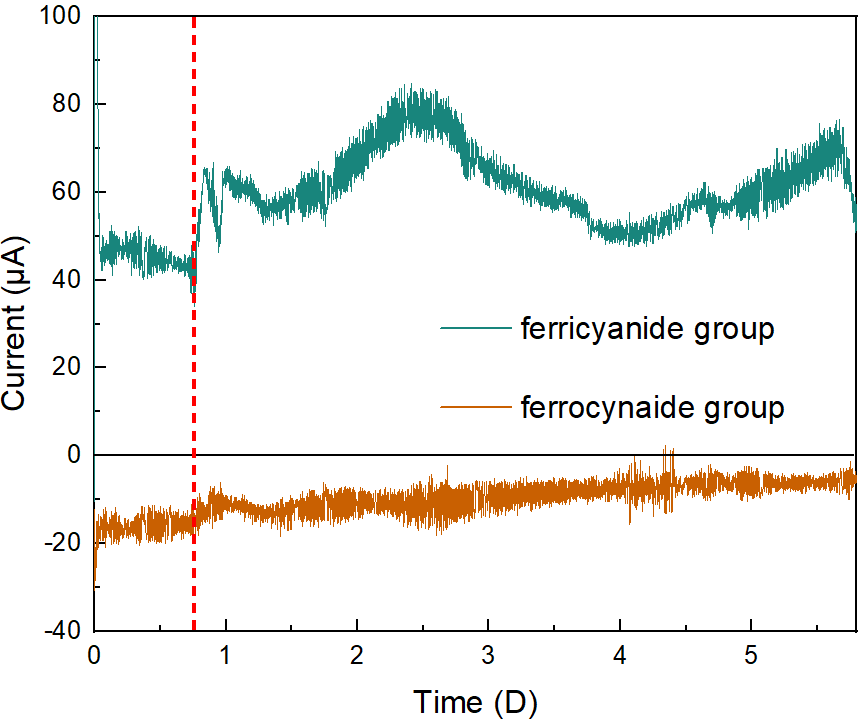


**Fig. S3.** Average currents of ferricyanide and ferrocyanide groups during 5 days’ monitoring. Ferricyanide group represents the BPV systems run with 0.5 mM ferrocyanide addition as well as 0 V (vs. Ag/AgCl) potential bias applied, and Ferrocyanide group means the BPV systems with 0.5 mM ferrocyanide addition as well as 0 V (vs. Ag/AgCl) potential bias applied. The red vertical dash line stands for the inoculation time point. Before inoculation, the reactors ran for over 20 h with air bubbling to get whole system stable and gain the baseline signal. For ferricyanide added systems, the current increased right after the inoculation, then followed by a transient drop for 2.4 h, that is caused by the MIMS measurement conducted for carbon fixation rate and respiration rate determination, which exhausted the dissolved CO_2_ and change the light condition briefly, so the photocurrent was influenced a lot but restored immediately after measurement.


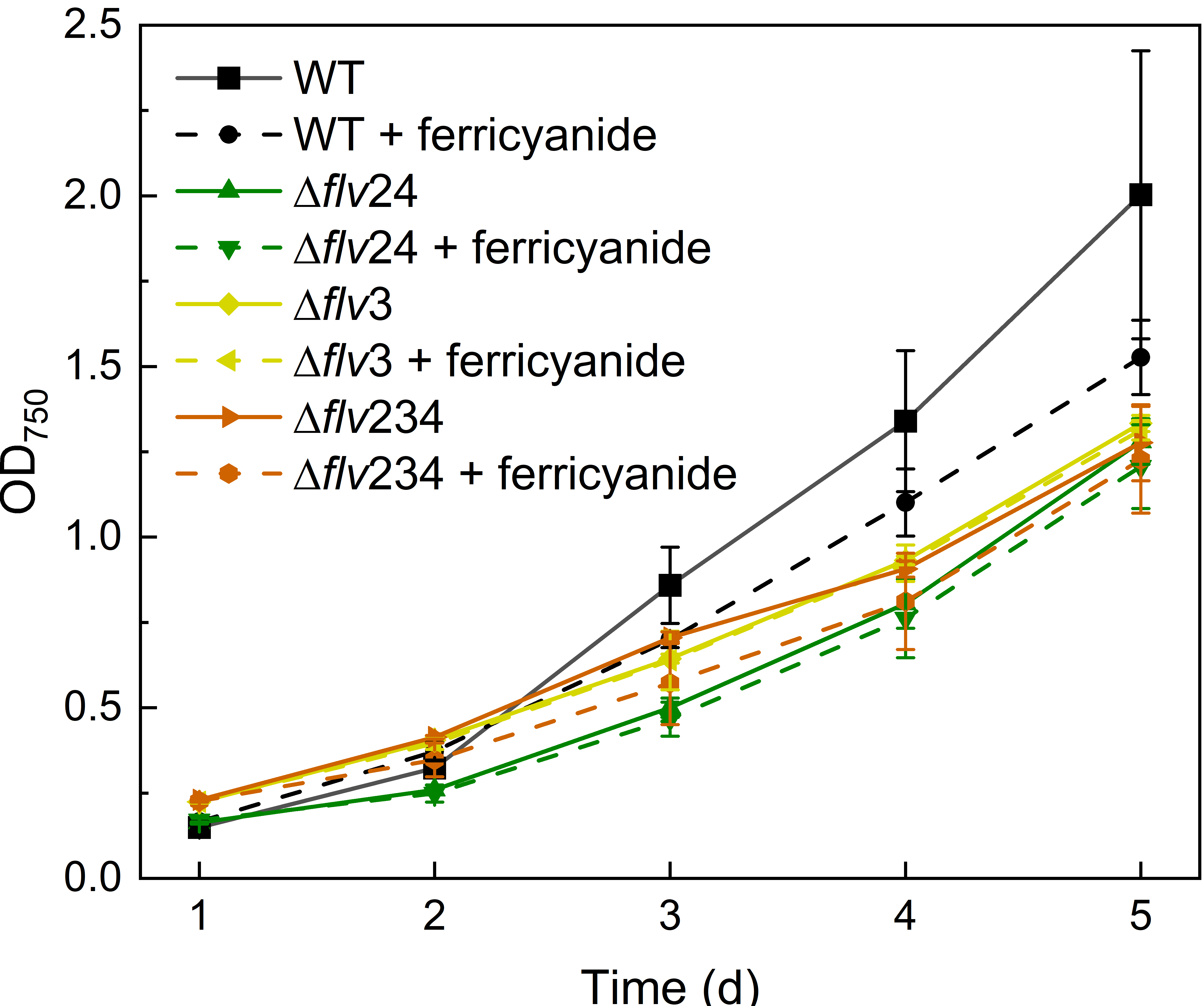


**Fig. S4.** Growth of 4 strains with or without ferricyanide addition during 4 days’ cultivation. Those cultures of trains with ferricyanide addition mean that 0.5 mM ferricyanide was introduced into the medium when inoculated. The error bars stand for the standard deviation over 3 replicates (n = 3).


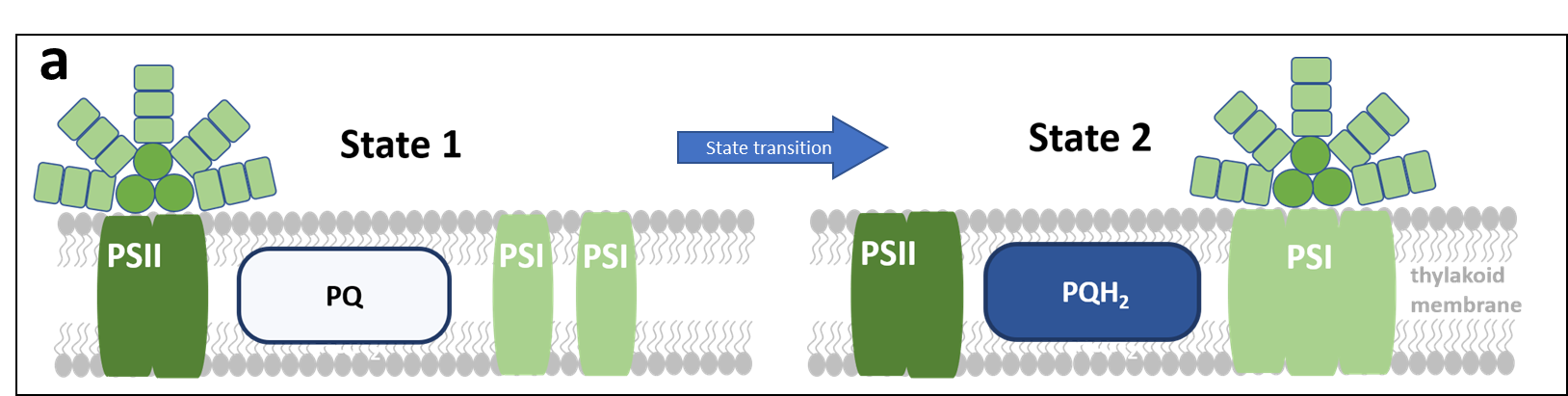


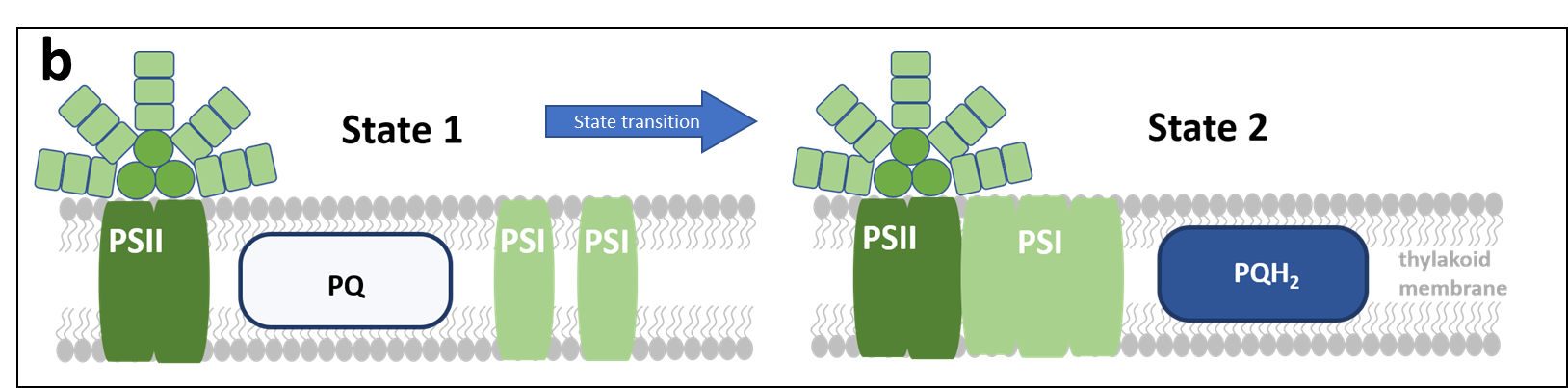


**Fig. S5.** Schematic diagram of state transition. (a) The phycobilisome movement model: In State 1, the phycobilisome is attached to PSII. When PQ pool becomes reduced, it shifts to State 2, where the phycobilisome moves to PSI. (b) The spillover model: Upon reduction of the PQ pool, the PSII-phycobilisome complex moves closer to PSI, facilitating energy spillover from PSII to PSI through their interaction.


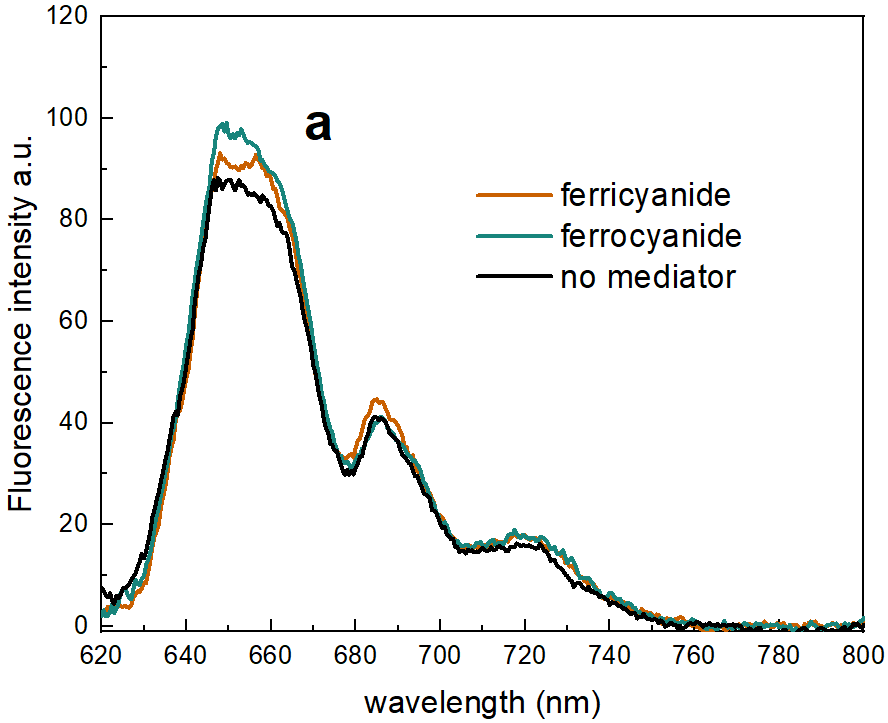

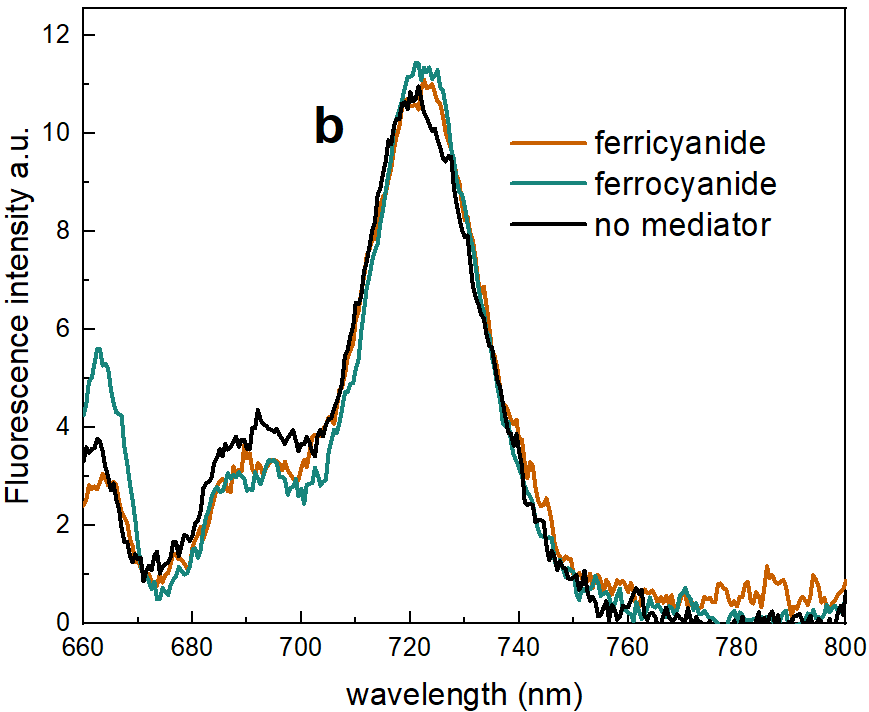


**Fig. S6.** 77K fluorescence spectra of culture symples from BPV systems, which was measured at an excitation wavelength of 580 nm (a) and 440 nm (b). In both plots, the fluorescence peaks at 650 nm is from PBS, the peaks at at 685 and 695 nm originate from PSII and the peak at 725 derives from PSI. ‘Ferricyanide’ represents the samples from BPV reactors with 0.5 mM ferricyanide addition as well as + 0.5 V (vs. Ag/AgCl) potential bias applied. ‘Ferrocyanide’ represents the samples from BPV reactors with 0.5 mM ferrocyanide addition as well as 0 V (vs. Ag/AgCl) potential bias. And ‘no mediator’ means the culture samples are from the blank BPV reactors.





**Fig. S7.** (a) P700 behavior with different concentration of KCN added. At 1000 ms, the red actinic light pulse of 1350 µmol photons·m^-2^·s^-1^ was applied and lasted for 600 ms. And positive value means oxidization of P700, whereas negative value represents reduction. (b) P700 redox change with various concentrations of ferricyanide in the culture. (c) P700 redox change with various concentrations of ferrocyanide. All measurements were conducted 16 times repeatedly with 30 s darkness intervals, the traces shown are the averages.


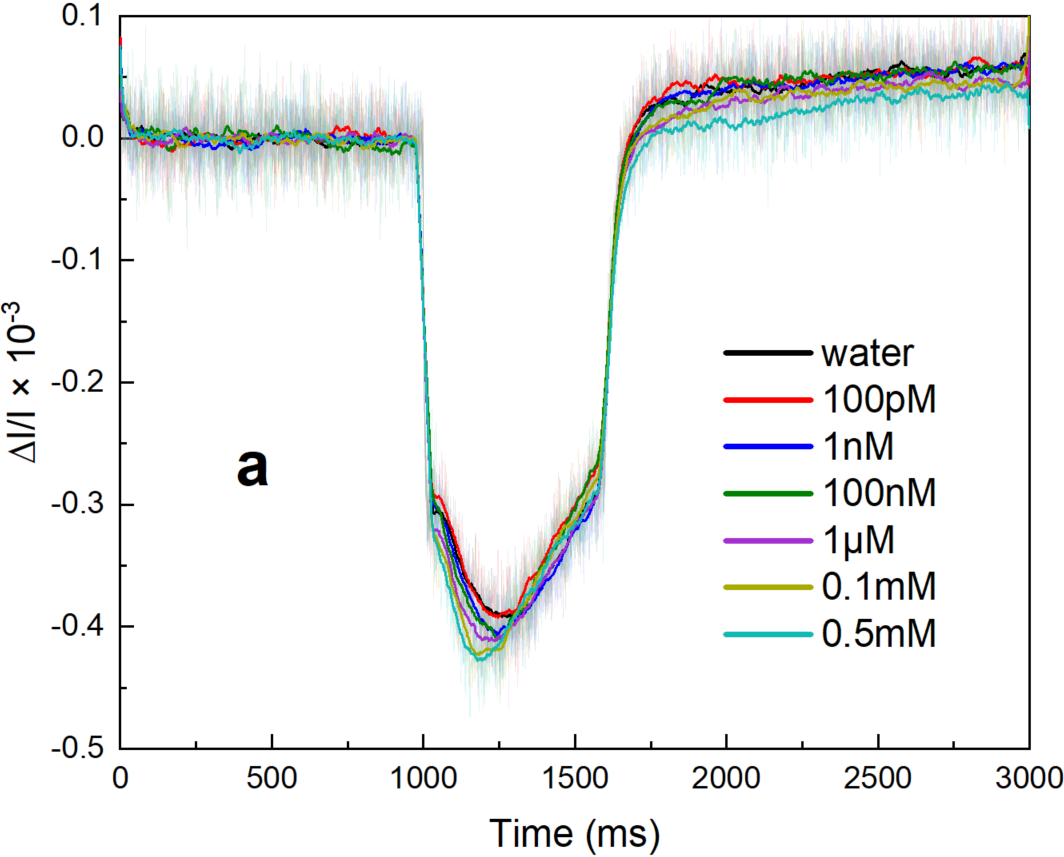

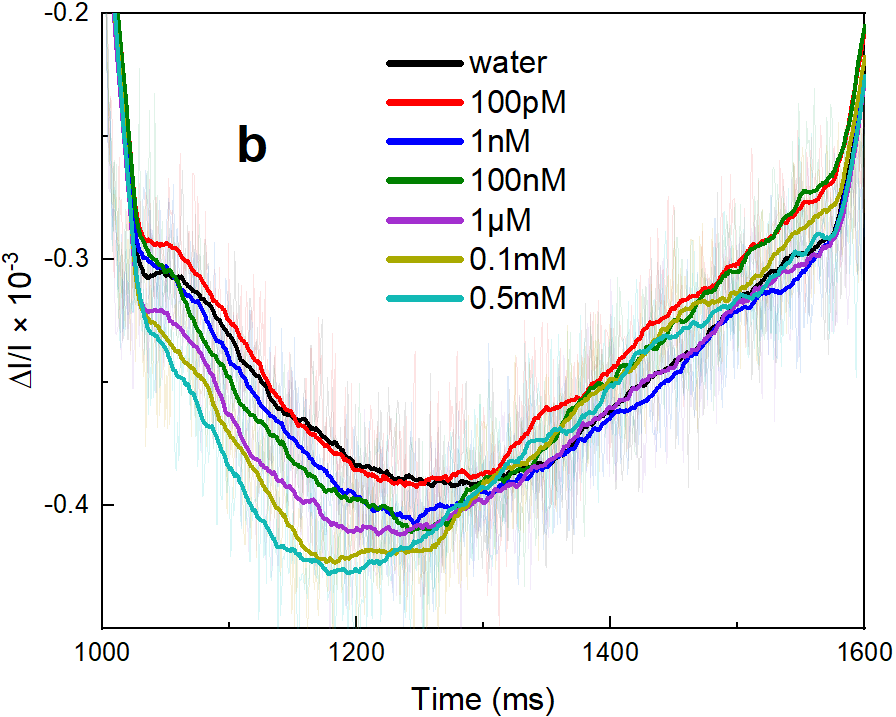

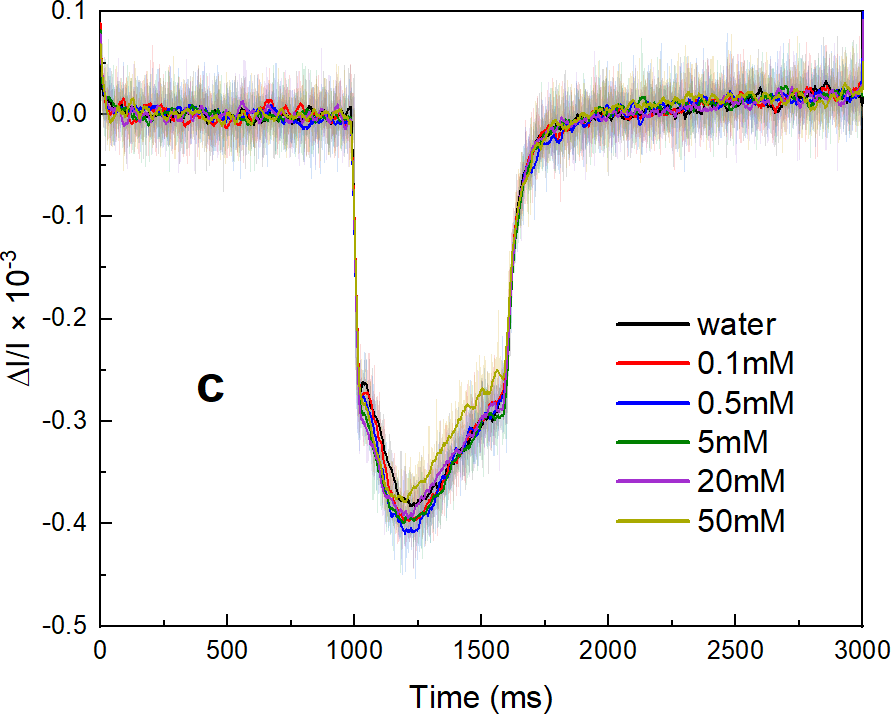

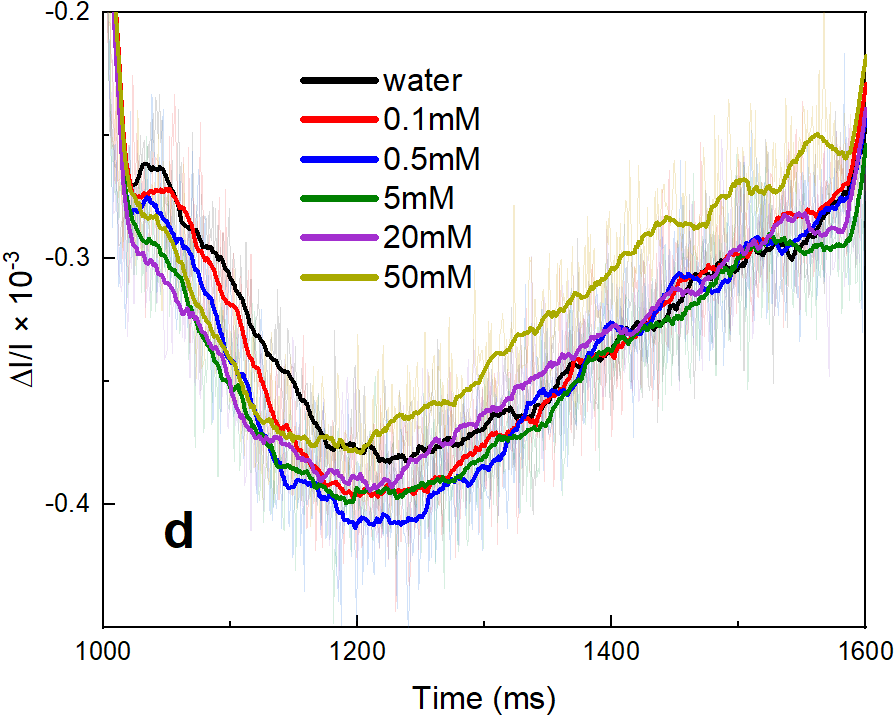


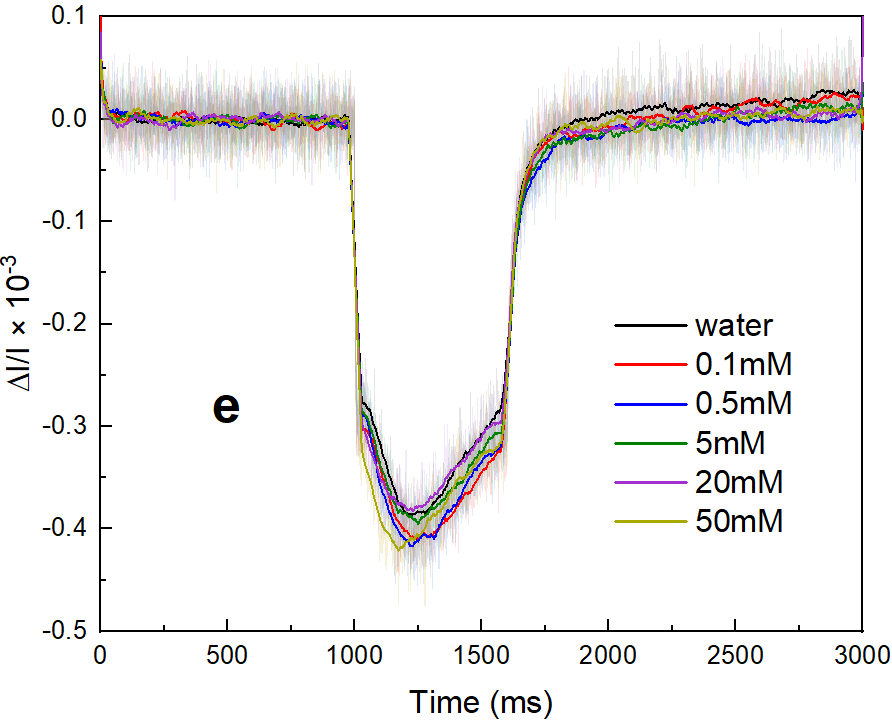

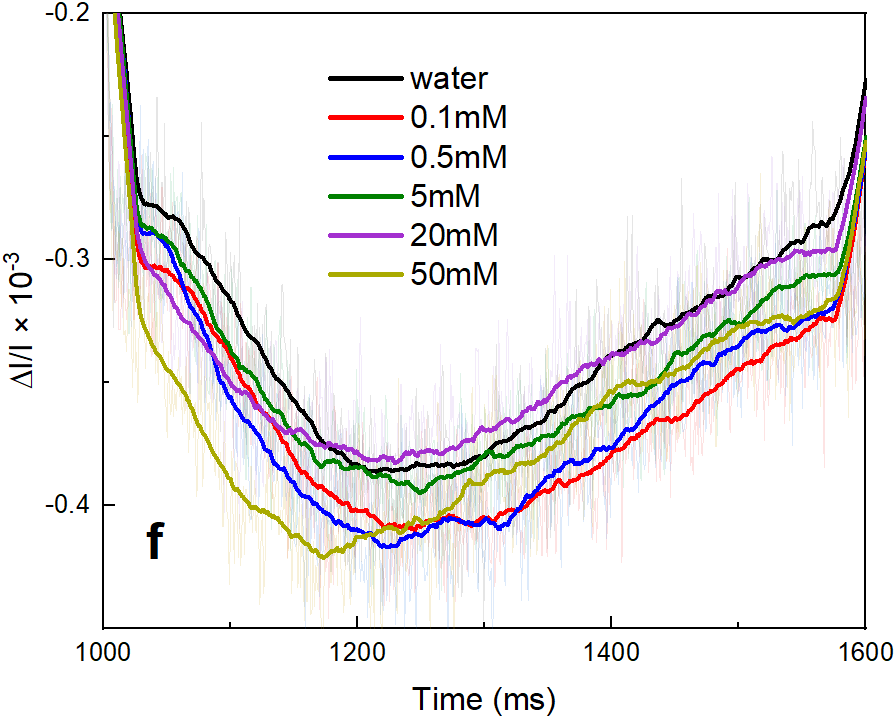


**Fig. S8.** Fd redox status dynamic change of *Synechocystis* at different concentrations of KCN and ferro/ferricyanide during 600 ms red light pulse. The redox change of Fd with (a) different concentration of KCN added, (c) different concentration of ferricyanide added and (e) different concentration of ferrocyanide added. (b) Local zoom of KCN added plot from 1000 to 1600 ms. (d), (f) Local zoom of ferricyanide and ferrocyanide addition plots from 1000 to 1600 ms, respectively. All measurements were conducted 16 times repeatedly with 30 s darkness intervals, the traces shown are the averages.

**Table S1** The energy transfer efficiency from PBS to PSI calculated based on 77K fluorescence spectra. The ratio of the fluorescence yield at 725 nm eission (F_PSI_) in the spectrum excited at 580 nm to that in 440 excited spectrum, i. e., F_PSI_ (580)/ F_PSI_ (440), can be considered as the PBS-PSI efficiency.

| sample from | ferricyanide group | ferrocyanide group | no mediator group |
| --- | --- | --- | --- |
| PBS-PSI efficiency | 1.56 | 1.684 | 1.47 |
